# 8-*O*-Acetyl-8-*epi*-9-de­oxygoniopypyrone

**DOI:** 10.1107/S1600536812010483

**Published:** 2012-03-17

**Authors:** Hoong-Kun Fun, Suchada Chantrapromma, Uma Prawat, Nawong Boonnak, Ibrahim Abdul Razak

**Affiliations:** a X-ray Crystallography Unit, School of Physics, Universiti Sains Malaysia, 11800 USM, Penang, Malaysia; b Crystal Materials Research Unit, Department of Chemistry, Faculty of Science, Prince of Songkla University, Hat-Yai, Songkhla 90112, Thailand; c Department of Chemistry, Faculty of Science and Technology, Phuket Rajabhat University, Muang, Phuket 83000, Thailand

## Abstract

The title compound (systematic name: 7-oxo-3-phenyl-2,6-dioxabicyclo­[3.3.1]nonan-4-yl acetate), C_15_H_16_O_5_, is a styryllactone derivative which was isolated from *Goniothalamus macrophyllus*. The mol­ecule has two fused rings consisting of a tetra­hydro-2*H*-pyran and a lactone ring, with the benzene ring and the acetyl group attached to the tetra­hydro-2*H*-pyran ring. The tetra­hydro-2*H*-pyran ring is in a standard chair conformation, whereas the lactone ring is in an envelope conformation. In the crystal, mol­ecules are linked by weak C—H⋯O inter­actions into sheets parallel to the *ac* plane. Weak C—H⋯π inter­actions are also observed.

## Related literature

For ring conformations, see: Cremer & Pople (1975 ▶). For bond-length data, see: Allen *et al.* (1987 ▶). For background to *Goniothamus* plants and the bioactivity of styryllactone compounds, see: Abdul-Wahab *et al.* (2011 ▶); Goh *et al.* (1995 ▶); Jiang *et al.* (2011 ▶); Smitinand (2001 ▶); Wattanapiromsakul *et al.* (2005 ▶). For the stability of the temperature controller used in the data collection, see: Cosier & Glazer (1986 ▶).
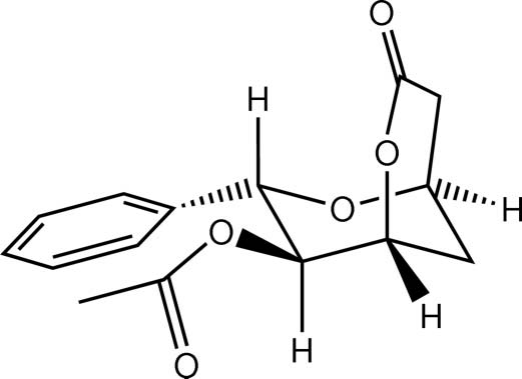


## Experimental

### 

#### Crystal data

C_15_H_16_O_5_
*M* *_r_* = 276.28
Monoclinic, 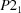

*a* = 10.1013 (3) Å
*b* = 5.7749 (2) Å
*c* = 11.2295 (3) Å
β = 95.207 (1)°
*V* = 652.36 (3) Å^3^
*Z* = 2
Mo *K*α radiation
μ = 0.11 mm^−1^
*T* = 100 K
0.59 × 0.43 × 0.43 mm

#### Data collection

Bruker APEXII CCD area-detector diffractometer
Absorption correction: multi-scan (*SADABS*; Bruker, 2009 ▶) *T*_min_ = 0.940, *T*_max_ = 0.956
24278 measured reflections
3105 independent reflections
3044 reflections with *I* > 2σ(*I*)
*R*_int_ = 0.027

#### Refinement

*R*[*F* ^2^ > 2σ(*F* ^2^)] = 0.027
*wR*(*F* ^2^) = 0.074
*S* = 1.08
3105 reflections
245 parameters
1 restraint
All H-atom parameters refined
Δρ_max_ = 0.36 e Å^−3^
Δρ_min_ = −0.21 e Å^−3^

### 

Data collection: *APEX2* (Bruker, 2009 ▶); cell refinement: *SAINT* (Bruker, 2009 ▶); data reduction: *SAINT*; program(s) used to solve structure: *SHELXTL* (Sheldrick, 2008 ▶); program(s) used to refine structure: *SHELXTL*; molecular graphics: *SHELXTL*; software used to prepare material for publication: *SHELXTL* and *PLATON* (Spek, 2009 ▶).

## Supplementary Material

Crystal structure: contains datablock(s) global, I. DOI: 10.1107/S1600536812010483/rz2717sup1.cif

Structure factors: contains datablock(s) I. DOI: 10.1107/S1600536812010483/rz2717Isup2.hkl

Supplementary material file. DOI: 10.1107/S1600536812010483/rz2717Isup3.cml

Additional supplementary materials:  crystallographic information; 3D view; checkCIF report

## Figures and Tables

**Table 1:** Hydrogen-bond geometry (Å, °) *Cg*1 is the centroid of the C8–C13 ring.

*D*—H⋯*A*	*D*—H	H⋯*A*	*D*⋯*A*	*D*—H⋯*A*
C5—H5⋯O2^i^	0.988 (15)	2.398 (15)	3.3563 (10)	163.2 (11)
C11—H11⋯O5^ii^	0.981 (16)	2.531 (16)	3.4986 (12)	168.9 (14)
C15—H15*A*⋯O5^iii^	0.99 (2)	2.43 (2)	3.4048 (12)	167 (2)
C2—H2*A*⋯*Cg*1^iv^	0.986 (16)	2.714 (15)	3.4619 (9)	133.0 (11)
C12—H12⋯*Cg*1^ii^	0.97 (2)	2.947 (18)	3.6566 (10)	130.9 (14)

Symmetry codes: (i) 
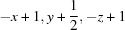
; (ii) 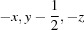; (iii) 
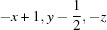
; (iv) 
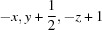
.

**Fig. 1.**
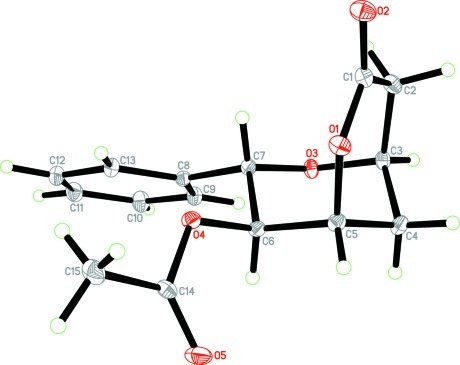
The molecular structure of the title compound, showing 50% probability displacement ellipsoids.

**Fig. 2.**
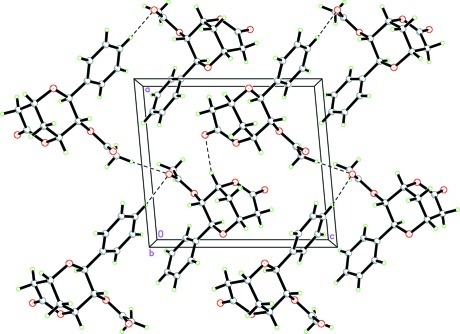
The crystal packing of the title compound viewed along the b axis, showing a sheet of molecules parallel to the ac plane. Weak C—H···O interactions are shown as dashed lines.

## supplementary crystallographic information

### Comment

*Goniothalamus macrophyllus*, a Thai medicinal plant, which is known in Thai as "Ching Dok Diao" belongs to the genus *Goniothalamus* (Smitinand, 2001). A study of methanolic extracts from the roots and stems of *G. macrophyllus* shows that the methanolic extracts exhibit good cytotoxicity against breast and lung carcinoma cancer cell lines with IC_50_ value in range 3.16-5.04 µg/mL (Wattanapiromsakul *et al.*, 2005). In addition, methanolic extract from the leaves of *G. umbrosus* showed antoxidant, antibacterial and antiviral activities (Abdul-Wahab *et al.*, 2011). The previous reports by Goh *et al.* (1995) and Jiang *et al.* (2011) showed that plants in *Goniothalamus* genus produce styryllactone compounds as major constituents and most of them exhibit potent cytotoxic activity. The above investigations has prompted us to search for cytotoxic components from *Goniothalamus* plants. Our research on bioactive compounds from *G. macrophyllus* yields the title compound (I), 8-*O*-Acetyl-8-*epi*-9-deoxygoniopypyrone. Herein the crystal structure of (I) was reported.

The molecule of (I) has a bicyclic skeleton (Fig. 1). The tetrahydro-2*H*-pyran ring (C3–C7/O3) is in a standard chair conformation whereas the lactone ring (C1–C5/O1/O2) adopts an envelope conformation with the puckering C4 atom having a deviation of 0.3806 (9) Å and puckering parameters *Q* = 0.5424 (9) Å, θ = 49.39 (9)° and φ = 234.03 (12)° (Cremer & Pople, 1975). The benzene ring is attached to the tetrahydro-2*H*-pyran ring at atom C7. The acetyl group is planar with the r.m.s. deviation of 0.0015 (1) Å for the four non-H atoms (C14/C15/O4/O5). The orientation of the acetyl group is described by the torsion angles C6–O4–C14–C15 = 175.73 (7)° and C6–O4–C14–O5 = -3.72 (12)°. The bond distances in (I) are within normal ranges (Allen *et al.*, 1987).

In the crystal packing (Fig. 2), the molecules are linked into sheets parallel to the *ac* plane by weak C—H···O interactions (Table 1). Weak C—H···π interactions (Table 1) are also observed.

### Experimental

The title compound was isolated from the methanolic extract of the *G. macrophyllus* by repeated column chromatography. Single crystals suitable for *X*-ray structure determination were obtained as colorless block-shaped crystals by slow evaporation of the solvent at room temperature after several days. M. p. 467–469 K.

### Refinement

All H atoms were located in difference maps and refined isotropically. A total of 2473 Friedel pairs were merged before final refinement as there is no large anomalous dispersion for the determination of the absolute configuration.

### Crystal data

**Table:**

C_15_H_16_O_5_	*F*(000) = 292
*M**_r_* = 276.28	*D*_x_ = 1.406 Mg m^−^^3^
Monoclinic, *P*2_1_	Melting point = 467–469 K
Hall symbol: P 2yb	Mo *K*α radiation, λ = 0.71073 Å
*a* = 10.1013 (3) Å	Cell parameters from 3105 reflections
*b* = 5.7749 (2) Å	θ = 1.8–35.0°
*c* = 11.2295 (3) Å	µ = 0.11 mm^−^^1^
β = 95.207 (1)°	*T* = 100 K
*V* = 652.36 (3) Å^3^	Block, colourless
*Z* = 2	0.59 × 0.43 × 0.43 mm

### Data collection

**Table:**

Bruker APEXII CCD area-detector diffractometer	3105 independent reflections
Radiation source: sealed tube	3044 reflections with *I* > 2σ(*I*)
Graphite monochromator	*R*_int_ = 0.027
φ and ω scans	θ_max_ = 35.0°, θ_min_ = 1.8°
Absorption correction: multi-scan (*SADABS*; Bruker, 2009)	*h* = −16→16
*T*_min_ = 0.940, *T*_max_ = 0.956	*k* = −9→9
24278 measured reflections	*l* = −18→17

### Refinement

**Table:**

Refinement on *F*^2^	Primary atom site location: structure-invariant direct methods
Least-squares matrix: full	Secondary atom site location: difference Fourier map
*R*[*F*^2^ > 2σ(*F*^2^)] = 0.027	Hydrogen site location: inferred from neighbouring sites
*wR*(*F*^2^) = 0.074	All H-atom parameters refined
*S* = 1.08	*w* = 1/[σ^2^(*F*_o_^2^) + (0.0526*P*)^2^ + 0.0379*P*] where *P* = (*F*_o_^2^ + 2*F*_c_^2^)/3
3105 reflections	(Δ/σ)_max_ = 0.001
245 parameters	Δρ_max_ = 0.36 e Å^−^^3^
1 restraint	Δρ_min_ = −0.21 e Å^−^^3^

### Special details

**Table:**

Experimental. The crystal was placed in the cold stream of an Oxford Cryosystems Cobra open-flow nitrogen cryostat (Cosier & Glazer, 1986) operating at 120.0 (1) K.
Geometry. All esds (except the esd in the dihedral angle between two l.s. planes) are estimated using the full covariance matrix. The cell esds are taken into account individually in the estimation of esds in distances, angles and torsion angles; correlations between esds in cell parameters are only used when they are defined by crystal symmetry. An approximate (isotropic) treatment of cell esds is used for estimating esds involving l.s. planes.
Refinement. Refinement of F^2^ against ALL reflections. The weighted R-factor wR and goodness of fit S are based on F^2^, conventional R-factors R are based on F, with F set to zero for negative F^2^. The threshold expression of F^2^ > 2sigma(F^2^) is used only for calculating R-factors(gt) etc. and is not relevant to the choice of reflections for refinement. R-factors based on F^2^ are statistically about twice as large as those based on F, and R- factors based on ALL data will be even larger.

### Fractional atomic coordinates and isotropic or equivalent isotropic displacement parameters (Å^2^)

**Table:**

	*x*	*y*	*z*	*U*_iso_*/*U*_eq_	
O1	0.38218 (6)	0.66249 (12)	0.48754 (5)	0.01367 (11)	
O2	0.33277 (7)	0.47946 (14)	0.64890 (6)	0.01839 (13)	
O3	0.07398 (6)	0.88291 (11)	0.39772 (5)	0.01228 (10)	
O4	0.31111 (6)	0.52721 (11)	0.25383 (5)	0.01305 (11)	
O5	0.43199 (7)	0.73105 (15)	0.13134 (6)	0.02024 (13)	
C1	0.30578 (8)	0.63582 (15)	0.57915 (7)	0.01278 (13)	
C2	0.19378 (8)	0.80331 (15)	0.59457 (7)	0.01390 (13)	
H2A	0.2199 (15)	0.887 (3)	0.6695 (13)	0.021 (4)*	
H2B	0.1169 (15)	0.716 (4)	0.6069 (13)	0.021 (4)*	
C3	0.16268 (8)	0.97661 (14)	0.49384 (7)	0.01248 (12)	
H3	0.1154 (14)	1.096 (3)	0.5230 (13)	0.016 (3)*	
C4	0.29247 (8)	1.05337 (15)	0.44717 (8)	0.01427 (13)	
H4B	0.3513 (16)	1.123 (4)	0.5091 (14)	0.024 (4)*	
H4A	0.2808 (16)	1.162 (3)	0.3857 (13)	0.021 (4)*	
C5	0.35311 (7)	0.83992 (14)	0.39615 (7)	0.01247 (13)	
H5	0.4394 (15)	0.875 (3)	0.3652 (12)	0.015 (3)*	
C6	0.25786 (7)	0.73974 (14)	0.29598 (7)	0.01126 (12)	
H6	0.2460 (14)	0.846 (3)	0.2307 (12)	0.015 (3)*	
C7	0.12169 (7)	0.68308 (14)	0.33982 (6)	0.01064 (12)	
H7	0.1354 (14)	0.550 (3)	0.3951 (12)	0.015 (3)*	
C8	0.01788 (7)	0.61412 (14)	0.24012 (7)	0.01132 (12)	
C9	−0.09128 (8)	0.75649 (15)	0.20889 (7)	0.01458 (13)	
H9	−0.0986 (15)	0.900 (3)	0.2471 (13)	0.020 (3)*	
C10	−0.19036 (9)	0.68736 (18)	0.12145 (8)	0.01878 (15)	
H10	−0.2673 (16)	0.777 (4)	0.1031 (15)	0.027 (4)*	
C11	−0.18009 (9)	0.47651 (18)	0.06303 (8)	0.01850 (15)	
H11	−0.2496 (16)	0.427 (3)	0.0016 (14)	0.020 (3)*	
C12	−0.07031 (9)	0.33484 (17)	0.09223 (8)	0.01722 (14)	
H12	−0.0621 (17)	0.185 (4)	0.0544 (15)	0.029 (4)*	
C13	0.02716 (8)	0.40161 (15)	0.18141 (7)	0.01443 (13)	
H13	0.1009 (16)	0.305 (4)	0.2043 (15)	0.027 (4)*	
C14	0.40180 (8)	0.54736 (15)	0.17195 (7)	0.01314 (13)	
C15	0.45668 (10)	0.31577 (18)	0.14257 (8)	0.01938 (16)	
H15A	0.493 (2)	0.317 (5)	0.0635 (18)	0.049 (6)*	
H15B	0.392 (2)	0.209 (5)	0.1444 (19)	0.056 (7)*	
H15C	0.522 (2)	0.284 (6)	0.201 (2)	0.064 (7)*	

### Atomic displacement parameters (Å^2^)

**Table:**

	*U*^11^	*U*^22^	*U*^33^	*U*^12^	*U*^13^	*U*^23^
O1	0.0109 (2)	0.0165 (3)	0.0139 (2)	0.0029 (2)	0.00278 (17)	0.0007 (2)
O2	0.0182 (3)	0.0198 (3)	0.0173 (3)	0.0043 (2)	0.0025 (2)	0.0040 (2)
O3	0.0107 (2)	0.0131 (2)	0.0130 (2)	0.00175 (19)	0.00023 (17)	−0.00312 (19)
O4	0.0137 (2)	0.0117 (2)	0.0146 (2)	0.00091 (19)	0.00635 (18)	−0.00016 (19)
O5	0.0234 (3)	0.0190 (3)	0.0200 (3)	0.0015 (2)	0.0110 (2)	0.0048 (2)
C1	0.0109 (3)	0.0152 (3)	0.0123 (3)	0.0010 (2)	0.0008 (2)	−0.0015 (2)
C2	0.0130 (3)	0.0168 (3)	0.0120 (3)	0.0034 (3)	0.0024 (2)	−0.0010 (3)
C3	0.0118 (3)	0.0123 (3)	0.0133 (3)	0.0010 (2)	0.0007 (2)	−0.0025 (2)
C4	0.0137 (3)	0.0115 (3)	0.0178 (3)	−0.0015 (2)	0.0023 (2)	−0.0022 (3)
C5	0.0105 (3)	0.0126 (3)	0.0145 (3)	−0.0006 (2)	0.0024 (2)	0.0000 (2)
C6	0.0107 (3)	0.0109 (3)	0.0126 (3)	0.0000 (2)	0.0034 (2)	−0.0004 (2)
C7	0.0100 (3)	0.0107 (3)	0.0115 (3)	0.0002 (2)	0.0024 (2)	−0.0007 (2)
C8	0.0110 (3)	0.0116 (3)	0.0116 (3)	−0.0010 (2)	0.0019 (2)	−0.0005 (2)
C9	0.0135 (3)	0.0149 (3)	0.0150 (3)	0.0011 (3)	−0.0007 (2)	−0.0014 (3)
C10	0.0162 (3)	0.0211 (4)	0.0181 (3)	0.0012 (3)	−0.0037 (3)	−0.0023 (3)
C11	0.0177 (3)	0.0219 (4)	0.0154 (3)	−0.0034 (3)	−0.0014 (3)	−0.0021 (3)
C12	0.0198 (3)	0.0162 (3)	0.0156 (3)	−0.0035 (3)	0.0011 (2)	−0.0034 (3)
C13	0.0153 (3)	0.0128 (3)	0.0151 (3)	−0.0005 (3)	0.0010 (2)	−0.0021 (2)
C14	0.0127 (3)	0.0163 (3)	0.0108 (3)	0.0021 (3)	0.0031 (2)	−0.0002 (3)
C15	0.0225 (4)	0.0185 (4)	0.0180 (4)	0.0062 (3)	0.0065 (3)	−0.0017 (3)

### Geometric parameters (Å, º)

**Table:**

O1—C1	1.3498 (10)	C6—C7	1.5377 (10)
O1—C5	1.4610 (10)	C6—H6	0.955 (16)
O2—C1	1.2101 (11)	C7—C8	1.5161 (11)
O3—C7	1.4295 (10)	C7—H7	0.989 (16)
O3—C3	1.4440 (10)	C8—C9	1.3945 (11)
O4—C14	1.3605 (9)	C8—C13	1.4003 (12)
O4—C6	1.4374 (10)	C9—C10	1.3953 (12)
O5—C14	1.2048 (11)	C9—H9	0.938 (18)
C1—C2	1.5103 (11)	C10—C11	1.3915 (14)
C2—C3	1.5216 (12)	C10—H10	0.942 (19)
C2—H2A	0.986 (16)	C11—C12	1.3930 (13)
C2—H2B	0.946 (17)	C11—H11	0.981 (16)
C3—C4	1.5214 (11)	C12—C13	1.3935 (12)
C3—H3	0.914 (17)	C12—H12	0.97 (2)
C4—C5	1.5122 (12)	C13—H13	0.946 (18)
C4—H4B	0.962 (17)	C14—C15	1.4960 (13)
C4—H4A	0.932 (17)	C15—H15A	0.99 (2)
C5—C6	1.5262 (11)	C15—H15B	0.90 (3)
C5—H5	0.989 (15)	C15—H15C	0.90 (3)
			
C1—O1—C5	121.54 (6)	C7—C6—H6	109.2 (8)
C7—O3—C3	115.50 (6)	O3—C7—C8	107.98 (6)
C14—O4—C6	116.40 (6)	O3—C7—C6	108.79 (6)
O2—C1—O1	117.88 (7)	C8—C7—C6	113.48 (6)
O2—C1—C2	122.06 (7)	O3—C7—H7	112.0 (9)
O1—C1—C2	120.03 (7)	C8—C7—H7	107.9 (9)
C1—C2—C3	116.25 (6)	C6—C7—H7	106.7 (8)
C1—C2—H2A	105.5 (10)	C9—C8—C13	118.91 (7)
C3—C2—H2A	109.5 (11)	C9—C8—C7	120.60 (7)
C1—C2—H2B	108.0 (11)	C13—C8—C7	120.43 (7)
C3—C2—H2B	110.0 (10)	C8—C9—C10	120.57 (8)
H2A—C2—H2B	107.2 (13)	C8—C9—H9	119.9 (10)
O3—C3—C4	110.32 (6)	C10—C9—H9	119.5 (10)
O3—C3—C2	112.43 (7)	C11—C10—C9	120.18 (8)
C4—C3—C2	108.75 (6)	C11—C10—H10	118.3 (12)
O3—C3—H3	103.9 (9)	C9—C10—H10	121.4 (12)
C4—C3—H3	113.5 (10)	C10—C11—C12	119.66 (8)
C2—C3—H3	107.9 (10)	C10—C11—H11	120.5 (11)
C5—C4—C3	106.52 (7)	C12—C11—H11	119.8 (11)
C5—C4—H4B	111.8 (11)	C11—C12—C13	120.14 (8)
C3—C4—H4B	111.7 (9)	C11—C12—H12	121.2 (10)
C5—C4—H4A	107.2 (11)	C13—C12—H12	118.5 (10)
C3—C4—H4A	113.3 (10)	C12—C13—C8	120.51 (8)
H4B—C4—H4A	106.3 (16)	C12—C13—H13	121.4 (12)
O1—C5—C4	111.60 (6)	C8—C13—H13	118.1 (12)
O1—C5—C6	108.96 (6)	O5—C14—O4	122.70 (8)
C4—C5—C6	109.77 (6)	O5—C14—C15	126.31 (7)
O1—C5—H5	105.4 (9)	O4—C14—C15	111.00 (7)
C4—C5—H5	111.4 (10)	C14—C15—H15A	111.3 (17)
C6—C5—H5	109.6 (8)	C14—C15—H15B	108.9 (17)
O4—C6—C5	109.68 (6)	H15A—C15—H15B	111 (2)
O4—C6—C7	107.22 (6)	C14—C15—H15C	106 (2)
C5—C6—C7	111.54 (6)	H15A—C15—H15C	110.6 (19)
O4—C6—H6	108.6 (9)	H15B—C15—H15C	109 (2)
C5—C6—H6	110.4 (10)		
			
C5—O1—C1—O2	177.18 (7)	C3—O3—C7—C8	−178.45 (6)
C5—O1—C1—C2	−4.92 (11)	C3—O3—C7—C6	−54.89 (8)
O2—C1—C2—C3	−174.31 (8)	O4—C6—C7—O3	171.98 (6)
O1—C1—C2—C3	7.88 (11)	C5—C6—C7—O3	51.89 (8)
C7—O3—C3—C4	61.21 (8)	O4—C6—C7—C8	−67.80 (8)
C7—O3—C3—C2	−60.37 (8)	C5—C6—C7—C8	172.11 (6)
C1—C2—C3—O3	84.88 (8)	O3—C7—C8—C9	8.26 (10)
C1—C2—C3—C4	−37.59 (9)	C6—C7—C8—C9	−112.42 (8)
O3—C3—C4—C5	−60.54 (8)	O3—C7—C8—C13	−168.99 (7)
C2—C3—C4—C5	63.20 (8)	C6—C7—C8—C13	70.34 (9)
C1—O1—C5—C4	32.61 (10)	C13—C8—C9—C10	0.66 (12)
C1—O1—C5—C6	−88.77 (8)	C7—C8—C9—C10	−176.62 (8)
C3—C4—C5—O1	−61.42 (8)	C8—C9—C10—C11	−1.02 (13)
C3—C4—C5—C6	59.49 (8)	C9—C10—C11—C12	−0.05 (14)
C14—O4—C6—C5	−83.04 (8)	C10—C11—C12—C13	1.47 (14)
C14—O4—C6—C7	155.69 (6)	C11—C12—C13—C8	−1.83 (13)
O1—C5—C6—O4	−53.13 (7)	C9—C8—C13—C12	0.76 (12)
C4—C5—C6—O4	−175.62 (6)	C7—C8—C13—C12	178.05 (8)
O1—C5—C6—C7	65.50 (8)	C6—O4—C14—O5	−3.72 (12)
C4—C5—C6—C7	−56.99 (8)	C6—O4—C14—C15	175.73 (7)

### Hydrogen-bond geometry (Å, º)

*Cg*1 is the centroid of the C8–C13 ring.

**Table:**

*D*—H···*A*	*D*—H	H···*A*	*D*···*A*	*D*—H···*A*
C5—H5···O2^i^	0.988 (15)	2.398 (15)	3.3563 (10)	163.2 (11)
C11—H11···O5^ii^	0.981 (16)	2.531 (16)	3.4986 (12)	168.9 (14)
C15—H15*A*···O5^iii^	0.99 (2)	2.43 (2)	3.4048 (12)	167 (2)
C2—H2*A*···*Cg*1^iv^	0.986 (16)	2.714 (15)	3.4619 (9)	133.0 (11)
C12—H12···*Cg*1^ii^	0.97 (2)	2.947 (18)	3.6566 (10)	130.9 (14)

Symmetry codes: (i) −*x*+1, *y*+1/2, −*z*+1; (ii) −*x*, *y*−1/2, −*z*; (iii) −*x*+1, *y*−1/2, −*z*; (iv) −*x*, *y*+1/2, −*z*+1.

## References

[bb1] Abdul-Wahab, N.-Z., Shahar, S., Abdullah-Sani, H., Pihie, A. H. L. & Ibrahim, N. (2011). *Afr. J. Microbiol. Res.* **5**, 3138–3143.

[bb2] Allen, F. H., Kennard, O., Watson, D. G., Brammer, L., Orpen, A. G. & Taylor, R. (1987). *J. Chem. Soc. Perkin Trans. 2*, pp. S1–19.

[bb3] Bruker (2009). *APEX2*, *SAINT* and *SADABS* Bruker AXS Inc., Madison, Wisconsin, USA.

[bb4] Cosier, J. & Glazer, A. M. (1986). *J. Appl. Cryst.* **19**, 105–107.

[bb5] Cremer, D. & Pople, J. A. (1975). *J. Am. Chem. Soc.* **97**, 1354–1358.

[bb6] Goh, S. H., Ee, G. C. L., Chuah, C. H. & Wei, C. (1995). *Aust. J. Chem.* **48**, 199–205.

[bb7] Jiang, M.-M., Feng, Y.-F., Gao, H., Zhang, X., Tang, J.-S. & Yao, X.-S. (2011). *Fitoterapia*, **82**, 524–527. 10.1016/j.fitote.2010.11.014 21075179

[bb8] Sheldrick, G. M. (2008). *Acta Cryst.* A**64**, 112–122. 10.1107/S0108767307043930 18156677

[bb9] Smitinand, T. (2001). *Thai Plant Names*, pp. 260–261. Bangkok: Prachachon Publisher.

[bb10] Spek, A. L. (2009). *Acta Cryst.* D**65**, 148–155. 10.1107/S090744490804362X PMC2631630 19171970

[bb12] Wattanapiromsakul, C., Wangsintaweekul, B., Sangprapan, P., Itharat, A. & Keawpradub, N. (2005). *Songklanakarin J. Sci. Technol* **27**, 480–487.

